# Using the predictive model of difficult endotracheal intubation to examine different simulators for airway management training: a pilot cross-sectional observational study

**DOI:** 10.1186/s12909-025-07413-2

**Published:** 2025-06-06

**Authors:** Ching-Hsiang Yu, En-Chih Liao, Yat-Pang Chau, Ming-Kun Huang, Ching-Yi Shen, Ding-Kuo Chien

**Affiliations:** 1 Department of Emergency Medicine, MacKay Memorial Hospital, Taipei, Taiwan; 2 Department of Medicine, MacKay Medical College, Section 2, Chungshan North Road, New Taipei City, Taiwan; 3 Institute of Biomedical Sciences, MacKay Medical College, New Taipei City, Taiwan

**Keywords:** Difficult endotracheal intubation, Emergency department, Medical education, Training model, Simulator, Manikin

## Abstract

**Background:**

In recent years, Taiwan’s medical education has increasingly emphasized simulated learning, particularly through advanced manikins designed for procedural training, including endotracheal intubation. Although key indicators and predictive techniques for assessing complexity have been documented, their use in evaluating these manikins remains notably lacking.The aim of this study was to appraise the potential association between our devised scoring system and the actual outcome of intubation procedures. Subsequently, this scoring system could potentially serve as an objective yardstick for quantifying the intricacy of training simulators.

**Methods:**

Nineteen post-graduate or emergency medicine trainees participated in this study. Intubation training involved four manikins, each with varying difficulty scores based on neck circumference, thyromental distance, airway obstruction, and Mallampati grade 3/4. Training modules included standard, advanced, and small adult intubation. Independent variables were training models and operator skill levels, while dependent variables included intubation time, success rate, tooth injury, gastric insufflation, uninflated cuff mishaps, perceived difficulty (rated 1–5), and laryngoscopy view quality (rated 1–4).

**Results and discussion:**

Intubation parameters were recorded for comparison across subgroups. Mean intubation times for models A, B, and D were 42.67 ± 15.32 *seconds*, 48.73 ± 17.54 *seconds*, and 50.22 ± 18.40 *seconds*, with success rates of 89.47%, 78.95%, and 68.42%, respectively. Model ‘C’ had the highest difficulty score (4.430 *points*), the longest intubation time (58.84 ± 22.63 *seconds*, *P* <.001), and the lowest success rate (57.89%, *P* <.001), and was rated most difficult by participants. Notably, subsequent intubation attempts showed reduced time and complexity compared to the initial one.In conclusions, our devised scoring metric demonstrated a remarkable congruence with the tangible outcomes of the challenging endotracheal intubation training model. This outcome lends credence to the potential applicability of our formula not only in assessing the intricacy of existing models but also as a guiding benchmark for the innovation and refinement of novel training manikins.

**Supplementary Information:**

The online version contains supplementary material available at 10.1186/s12909-025-07413-2.

## Background

Ensuring adequate oxygenation in emergency and critically ill patients is a fundamental responsibility for airway healthcare practitioners [1]. However, endotracheal intubation can be particularly challenging for medical students and junior residents due to its complexity, the absence of a universally standardized algorithm, and limited clinical exposure [2, 3]. To address these challenges, medical education in Taiwan has increasingly incorporated simulation-based training, which has been shown to improve proficiency in invasive procedures, including endotracheal intubation [4].

Despite the benefits of simulation-based training, the effectiveness of airway management simulators may be influenced by anatomical differences between populations. Most existing studies on airway management devices and training models have been conducted in predominantly Caucasian populations, raising concerns about their applicability to Asian patients due to anatomical variations [5].Previous research on obstructive sleep apnea (OSA) has highlighted significant differences in upper airway anatomy between Asian and Caucasian populations, emphasizing the need for population-specific considerations in airway management [6].

Several predictive models have been developed to assess the difficulty of endotracheal intubation in clinical settings, with some models specifically designed for Asian populations [7, 8]. However, these predictive models have not been systematically utilized to evaluate the suitability of different simulation models for airway management training.

In our previous study, we developed a predictive model incorporating four key variables—body mass index (BMI), thyromental distance, upper airway obstruction, and Mallampati classification—to assess intubation difficulty [9]. This model demonstrated strong predictive performance, with a sensitivity of 79.5% and a specificity of 81.7%.

The aim of this study is to examine the applicability of our predictive model in assessing the suitability of different airway management simulators. Specifically, we seek to determine whether the intubation difficulty predicted by our model aligns with actual intubation experiences in a simulated training setting. By addressing this gap in the literature, our study provides insights into the effectiveness of existing airway training models and their relevance to diverse patient populations.

## Methodology and methods

### Evaluation of the normal and difficult training models for intubation

**Fig. 1:**
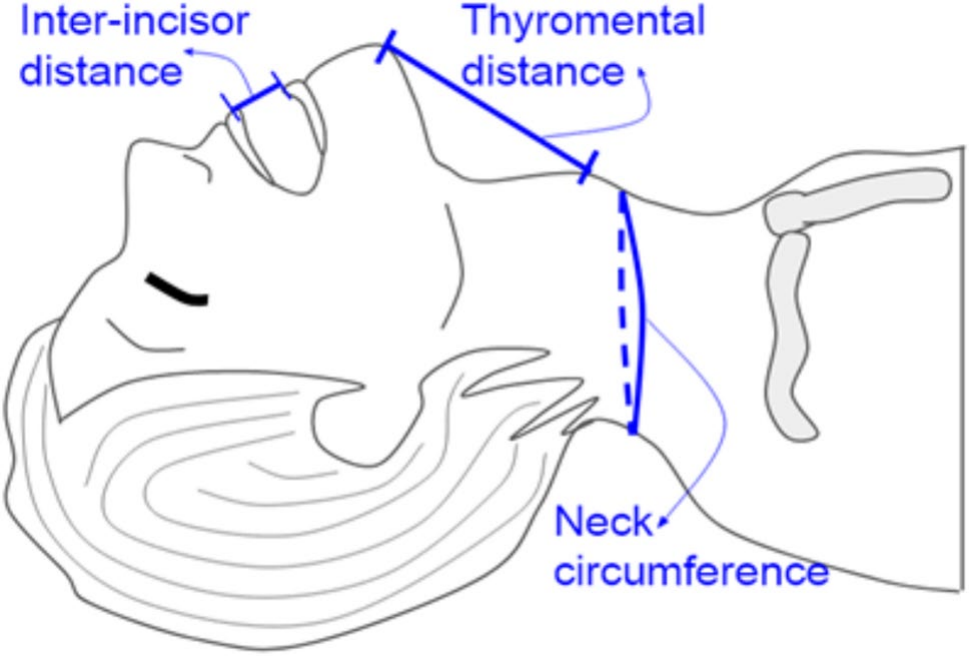
The definitions of inter-incisor distance, thyromental distance, and neck circumference. The distance between the upper and lower incisors with the mouth opened the widest was defined as the inter-incisor distance, and the distance between the prominent point of the mentum to the thyroid notch was the thyromental distance, the neck circumference was measured below the laryngeal prominence and at the level of the mid-cervical spine

To substantiate the authenticity of our prognosticative model and intricate formula devised for anticipating intricate intubation scenarios, we deliberately selected a quartet of simulated models tailor-made for intubation exercises. A total of five different intricate intubation training models were selected for training purposes, including Model A for standard adults, Model B, C, and D for advanced adults, as well as Model D represents a small adult airway, specifically designed to facilitate training in more challenging airway management scenarios. This model was selected for our study because it provides a higher level of difficulty, making it ideal for testing trainees’ proficiency in difficult airway situations. Detailed information on brands, models, and specifications, including Mallampati score, inter-incisor distance, thyromental distance, neck circumference, and estimated BMI, is listed in Table 1. These models have garnered widespread employment within the realms of medical pedagogy and emergency personnel training. Subsequently, we undertook the meticulous measurement of several anatomical dimensions, encompassing inter-incisor span, thyromental distance, and neck circumference (Fig. 1). Furthermore, we documented the Mallampati score and the Cormack & Lehane classification. To estimate BMI, we employed a calculation entailing neck circumference multiplied by a factor of 0.7. This approach was rooted in the precedent set by previous investigations, wherein the Beta coefficient establishing the relationship between neck circumference and BMI resided within the range of 0.6 to 0.8. As a culmination of our methodology, we proceeded to compute the intricate score about arduous endotracheal intubation. This intricate score was derived utilizing the formula outlined in the ensuing discourse:

**Table 1:**
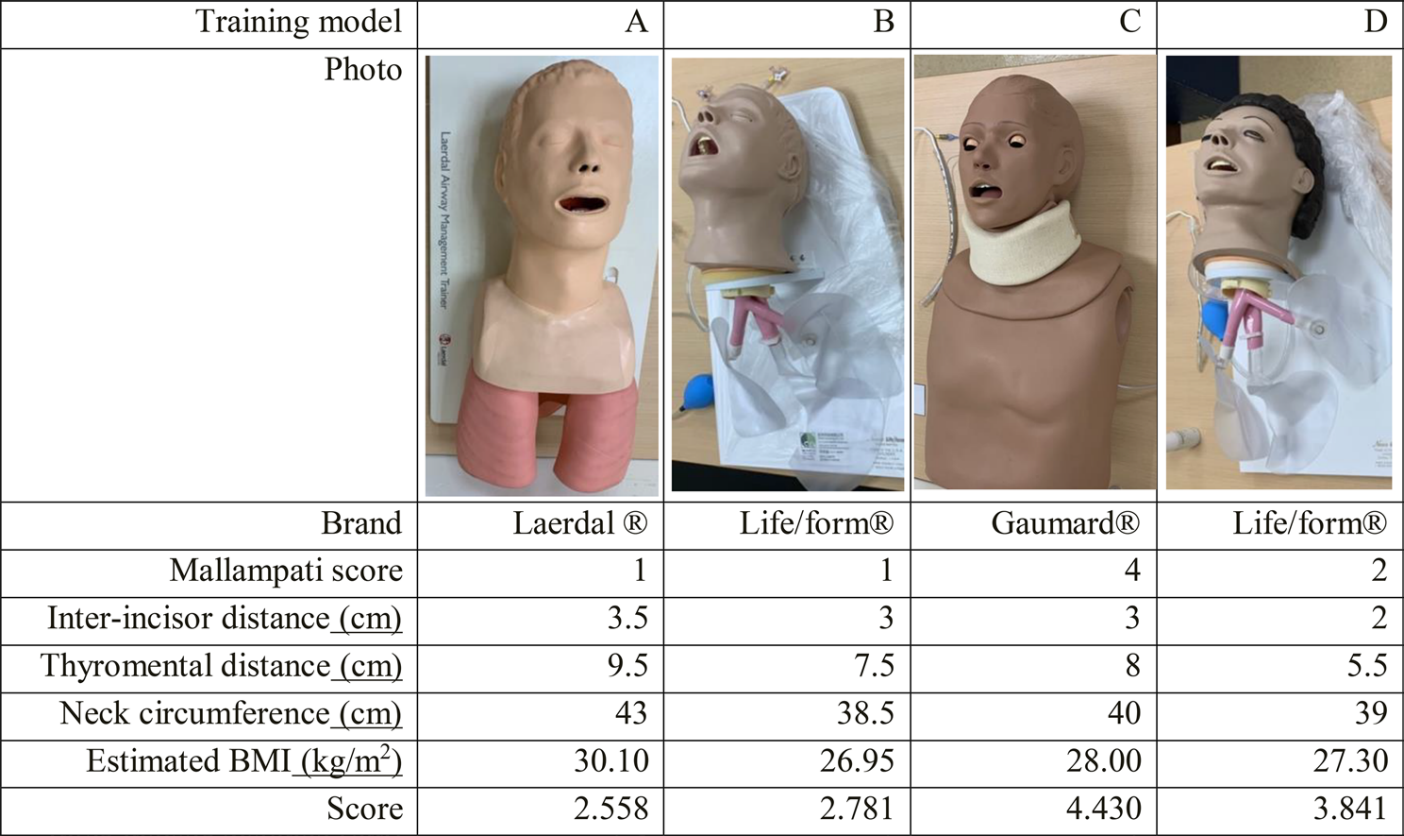
The characteristics of the four airway training models

Score for difficult endotracheal intubation = (neck circumference * 0.7 * 0.239) + (thyromental distance * -0.488) + (upper airway obstruction * 1.396) + (Mallampati grade 3/4 * 1.642).

### Study subjects and process of endotracheal intubation test

This was a cross-sectional study designed to evaluate different simulators for airway management training among emergency medicine residents and post-graduate year (PGY) doctors. Emergency medicine is a board-certified specialty under Taiwan’s Ministry of Health and Welfare. The study was conducted in the emergency department of a tertiary care hospital. Data collection took place during a simulation-based training session on April 8, 2021.

Eligibility criteria included being currently enrolled in a post-graduate or emergency residency program. The study involved the use of four distinct simulation stations, each equipped with a unique airway management model of varying complexity. Esteemed visiting staff members from the emergency department acted as examiners. Prior to the examination, participants received a detailed briefing on the standard intubation procedure. Each participant performed intubation on each model twice, with a 90-second time limit for each attempt. The following parameters were meticulously recorded by the examiners:

1. Intubation time during the first attempt.
2. Success rate of the first intubation attempt.
3. Incidence of tooth injury during the initial endeavor.
4. Occurrences of gastric insufflation (stomach bagging) during the initial attempt.
5. Instances of uninflated cuff mishaps during the first attempt.
6. Perceived difficulty level before intubation (from 1 to 5).
7. Quality of laryngoscopy view obtained during the first attempt (from 1 to 4).
8. Perceived difficulty subsequent to the first intubation attempt.
9. Intubation time during the second attempt.
10. Success rate of the second intubation attempt.
11. Potential tooth injury during the second endeavor.
12. Occurrences of gastric insufflation during the second attempt.
13. Instances of uninflated cuff mishaps during the second attempt.
14. Quality of laryngoscopy view obtained during the second attempt (from 1 to 4).
15. Perceived difficulty after the second intubation attempt (from 1 to 5).

Data for each variable of interest were collected directly by the examiners during the simulation sessions. These data included objective measures such as intubation time and success rates, as well as subjective assessments like perceived difficulty and laryngoscopy view quality.

### Statistical analysis

Statistical Package for the Social Sciences (SPSS) version 24.0 (IBM, Armonk, NY, USA) and RStudio version 2023.12.1 + 402 with packages of “DescTools” and “FSA” were used for data management and statistical analysis [10–12]. Categorical variables were represented using frequency distributions and reported as n (%), and continuous variables were reported as means ± standard deviation. Kruskal-Wallis tests and Wilcoxon signed-rank tests were used to compare differences between two, or more than two independent/dependent groups, then Bonferroni tests were used as post hoc tests for the comparisons among more than two groups. Statistical significance was set by a *P* value less than 0.05.

## Results

Among the array of endotracheal intubation training models selected, encompassing the Laerdal^®^ (Stavanger, Norway)Airway Management Trainer (designated as model A), the Life/form^®^ (Nasco Healthcare, Fort Atkinson, WI, USA)Advanced Airway Larry Trainer Head with Stand (designated as model B), the Gaumard^®^(Miami, FL, USA) Adult Advanced Multipurpose Airway & Cardiopulmonary Resuscitation (CPR)Trainer (designated as model C), and the Life/form^®^ Adult Airway Management Trainer with Stand (designated as model D), a comprehensive collection of anatomical dimensions and physical observations were painstakingly documented, constituting Table 1.

All selected models exhibited a body mass index (BMI) of ≥ 25, indicative of overweight or obesity. Additionally, none of the models had upper airway obstruction. Model C had the highest calculated difficulty score (4.430), exceeding the threshold for difficult intubation (cut-off = 4), followed by Model D (3.841), Model B (2.781), and Model A (2.558).

A total of 19 physicians participated in the study, including two post-graduate year (PGY) doctors and 17 emergency medicine residents. Among the residents, four were in their first year (R1), five in their second year (R2), four in their third year (R3), and four in their fourth year (R4).

Table 2 compares fifteen intubation-related parameters across the four training models. The Kruskal-Wallis test was used to identify significant differences. The following parameters showed no statistically significant differences: uninflated cuff in the first attempt (*P* =.567), success rate of the second attempt (*P* =.052), stomach bagging in the second attempt (*P* =.143), and uninflated cuff in the second attempt (*P* =.288). Model C had a significantly longer intubation time in both attempts and a lower success rate on the first attempt. The laryngoscopic view grade and perceived difficulty were higher for Model C compared to other models. Additionally, Model C had a significantly higher stomach bagging rate in the first attempt compared to Model D (*P* =.032) and a significantly higher pre-intubation difficulty level compared to Models A and B (both P *<*.001). Conversely, Model A had the highest incidence of tooth injury in both the first and second attempts.

**Table 2:** Comparisons of intubation-related indicators among the four training models

Training model	A	B	C	D	*P* value
Intubation time of the first attempt	28.00 ± 9.10	26.37 ± 9.83	58.84 ± 22.63*	33.89 ± 13.06	< 0.001
Success rate of the first attempt	94.74%	94.74%	57.89%*	100.00%	< 0.001
Tooth injury on the first attempt	36.84%^*^	5.26%	0.00%	0.00%	< 0.001
Stomach bagging on the first attempt	5.26%	5.26%	26.32%^#^	0.00%	0.027
Uninflated cuff in the first attempt	5.26%	5.26%	0.00%	0.00%	0.567
Difficulty before intubation	2.68 ± 0.58	2.68 ± 0.75	4.05 ± 0.78^$^	3.32 ± 0.82	< 0.001
laryngoscopy view in the first attempt	1.79 ± 0.63	1.84 ± 0.83	3.05 ± 0.85*	1.89 ± 0.66	< 0.001
Difficulty after the first attempt	2.42 ± 1.07	2.16 ± 1.26	4.53 ± 0.70*	3.11 ± 1.05	< 0.001
Intubation time of the second attempt	24.84 ± 8.952	21.74 ± 9.41	55.47 ± 23.85*	25.53 ± 11.30	< 0.001
Success rate of the second attempt	94.74%	94.74%	68.42%	89.47%	0.052
Tooth injury on the second attempt	31.58%^*^	5.26%	0.00%	0.00%	< 0.002
Stomach bagging on the second attempt	5.26%	0.00%	21.05%	10.53%	0.143
Uninflated cuff in the second attempt	10.53%	5.26%	0.00%	0.00%	0.288
Laryngoscopy view in the second attempt	1.58 ± 0.61	1.53 ± 0.61	3.11 ± 0.74*	1.89 ± 0.81	< 0.001
Difficulty after the second attempt	2.00 ± 0.88	1.84 ± 1.07	4.37 ± 0.76*	2.58 ± 1.12	< 0.001

^*^P <.05 to all the other models. ^#^*P* <.05 to model D. ^$^*P* <.05 to model A and B

Table 3 compares fifteen intubation-related parameters across the five training levels. PGY doctors had a significantly higher stomach bagging rate during the first attempt compared to *R*2 and R3 (*P* =.021 and 0.029, respectively). The R4 group had a significantly higher rate of uninflated cuff incidents in the second attempt compared to R2 (*P* =.043).Seven intubation-associated parameters were analyzed across both intubation attempts using Wilcoxon-signed rank tests (Table 4). A statistically significant reduction was observed in both intubation time and perceived difficulty in the second attempt (*P* <.001 and *P* <.001, respectively).

**Table 3:** Comparisons of intubation-related indicators among different training levels of the operators

	PGY	R1	R2	R3	R4	*P* value
Intubation time of first attempt	46.88 ± 20.35	31.31 ± 13.19	34.90 ± 17.99	32.44 ± 19.31	43.88 ± 24.12	0.095
Success rate of first attempt	62.50%	87.50%	95.00%	93.75%	81.25%	0.175
Tooth injury in first attempt	25.00%	6.25%	5.00%	18.75%	6.25%	0.387
Stomach bagging on first attempt	37.50%^&^	12.50%	0.00%	0.00%	12.50%	0.021
Uninflated cuff in first attempt	0.00%	0.00%	5.00%	0.00%	6.25%	0.680
Difficulty before intubation	3.25 ± 0.71	3.25 ± 0.86	3.50 ± 1.05	2.81 ± 1.05	3.06 ± 0.68	0.315
laryngoscopy view in first attempt	2.25 ± 1.04	2.19 ± 0.91	2.15 ± 0.88	2.13 ± 0.89	2.06 ± 1.00	0.981
Difficulty after first attempt	3.38 ± 1.60	3.38 ± 0.96	2.90 ± 1.45	2.50 ± 1.32	3.31 ± 1.54	0.306
Intubation time of second attempt	46.38 ± 21.91	27.81 ± 12.77	29.35 ± 19.34	32.06 ± 22.05	31.75 ± 22.40	0.114
Success rate of second attempt	75.00%	81.25%	95.00%	100.00%	75.00%	0.142
Tooth injury in second attempt	25.00%	6.25%	5.00%	6.25%	12.50%	0.508
Stomach bagging on second attempt	25.00%	12.50%	0.00%	0.00%	18.75%	0.096
Uninflated cuff in second attempt	0.00%	0.00%	0.00%	0.00%	18.75%^+^	0.021
laryngoscopy view in second attempt	1.50 ± 0.76	2.13 ± 0.89	2.20 ± 1.06	1.94 ± 0.68	2.06 ± 1.12	0.483
Difficulty after second attempt	3.00 ± 1.41	3.13 ± 1.26	2.65 ± 1.46	2.44 ± 1.46	2.44 ± 1.37	0.500

^&^*P* <.05 between PGY and R2, PGY and R3. ^+^*P* <.05 between R2 and R4

**Table 4:** Comparisons of intubation-related indicators between first and second attempts

Intubation attempt	First attempt	Second attempt	*P* value
Intubation time	36.78 ± 19.48	31.89 ± 19.94	< 0.001*
Success rate	87.0%	87.0%	1.000
Tooth injury	11.0%	9.0%	0.790
Stomach bagging	9.2%	9.2%	1.000
Uninflated cuff	3.0%	4.0%	0.773
laryngoscopy view	2.14 ± 0.91	2.03 ± 0.94	0.143
Difficulty after attempt	3.05 ± 1.38	2.70 ± 1.39	< 0.001*

*P <.05

**Fig. 2:**
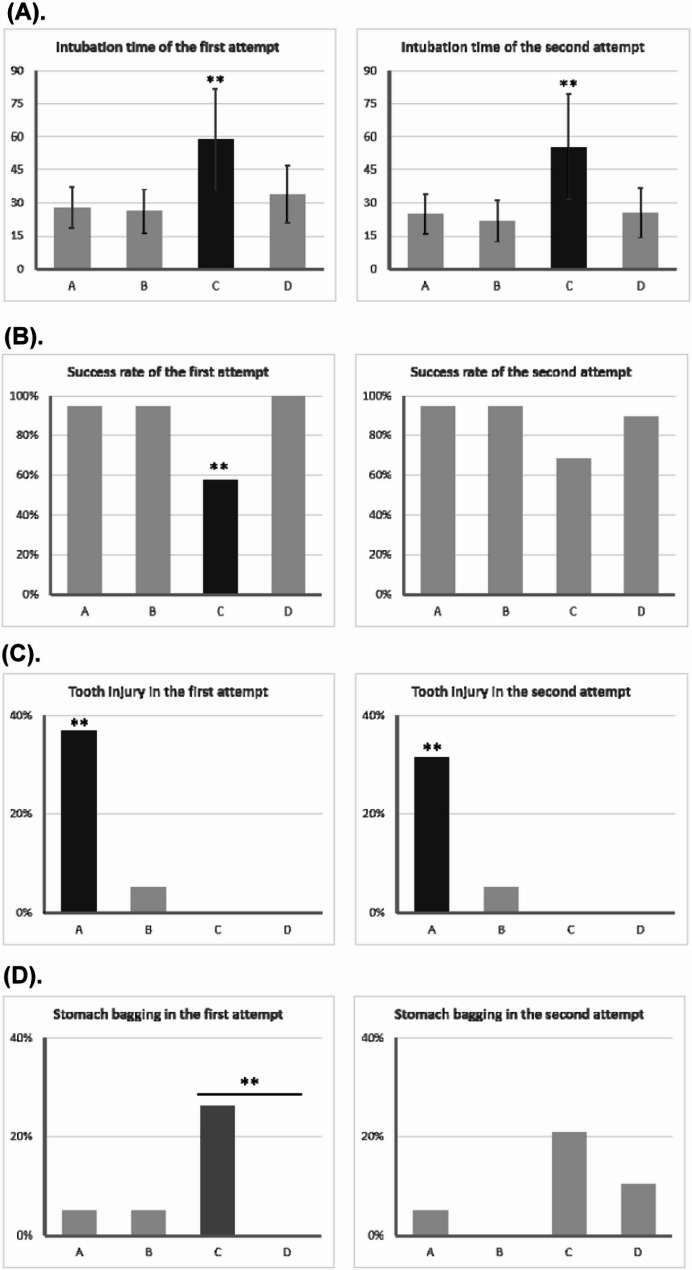
Comparisons of intubation-related indicators among 4 training models in the first and second attempt. (**A**) Intubation time. (**B**) Success rate. (**C**) Tooth injury. (**D**) Stomach bagging. * *P* <.05, ** *P* <.001

**Fig. 3:**
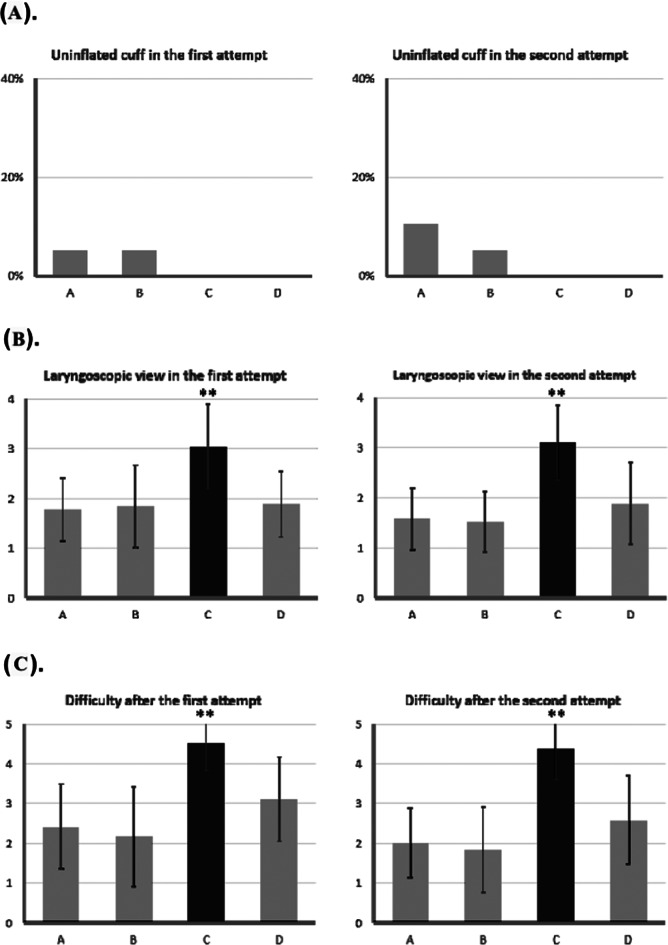
Comparisons of intubation-related indicators among 4 training models in the first and second attempt. (**A**) Uninflated cuff. (**B**) Laryngoscopy view. (**C**) Difficulty after attempt. * *P* <.05, ** *P* <.001

Figures 2 and 3 illustrate variations in intubation-associated indicators between the first and second attempts. Model C had the longest intubation time in both attempts, but a general trend of decreased intubation time in the second attempt was observed across all models. A similar trend was noted for perceived difficulty.

## Discussion

The study evaluated different simulators for airway management training by utilizing a predictive model of difficult endotracheal intubation. Among the four models (A, B, C, and D), Model C was the most challenging for intubation, exceeding the defined difficulty threshold. Significant differences were observed in intubation performance, particularly in terms of prolonged intubation duration and increased difficulty associated with Model C, while Model A had a higher incidence of tooth injury. Furthermore, differences in performance were noted between post-graduate year (PGY) doctors and senior residents, emphasizing the impact of training levels on intubation success. Our difficult intubation prediction scores correlated well with the actual testing results, supporting their utility in differentiating higher-order modules. Repeated intubation attempts led to improved performance, reinforcing the importance of structured and repeated training in airway management.

A constraint in designing a novel training model was the inability to assess the manikin’s BMI accurately. Due to material disparities, traditional BMI measurements were infeasible. Instead, we estimated BMI using neck circumference, based on prior research demonstrating a strong correlation between the two (Beta coefficient range: 0.62–0.83) [13, 14]. This approach allowed for a more realistic assessment of intubation difficulty and aligns with studies linking neck circumference to conditions such as obstructive sleep apnea [15, 16] and obesity [17–21].

Clinically, our findings have important implications for airway management training. Intubation time is widely used as an indicator of procedural success and patient outcomes [22, 23]. Our results emphasize that difficulty scores, in addition to time and success rates, are crucial metrics for evaluating training models. Interestingly, Model C was classified as the most challenging despite not having the highest BMI or shortest thyromental distance. This discrepancy highlights the importance of a multifactorial approach to assessing intubation difficulty rather than relying on individual anatomical predictors.

Prior studies have shown that simulation-based endotracheal intubation training improves success rates and reduces procedure time over time [24]. Our findings align with these results, suggesting that continued practice, even without specific interventions between training sessions, leads to skill enhancement and reduced perceived difficulty. This underscores the value of structured simulation-based learning in developing both technical proficiency and clinician confidence [25].

An ideal simulator should replicate clinical scenarios accurately [26]. Tooth injury is a recognized complication of intubation, particularly involving the upper incisors, which can have medicolegal consequences [27]. In our study, Model A uniquely incorporated an auditory alarm mechanism to signal tooth contact. Future simulator development could benefit from integrating pressure-sensitive bite apparatuses across multiple models to provide a more comprehensive evaluation of this risk [28].

Another key consideration is anatomical realism. The thyromental distances of our manikin models were longer than those observed in Chinese patient populations [9, 29]. Given prior research demonstrating racial differences in thyromental distance cut-off values for predicting difficult intubation [30, 31], future manikin designs for Chinese populations should adjust these dimensions accordingly.

Upper airway obstruction was incorporated into our predictive model but was not simulated in the manikins. Current airway training models do not typically include foreign body obstruction scenarios. However, integrating such a feature—similar to Laerdal’s Choking Charlie—could enhance training for complex airway management [32]. A manikin designed to simulate airway obstruction and removal techniques would provide valuable additional training opportunities [33].

This study has several limitations. First, our predictive model may be subject to parameter overcompensation. For example, the upper airway obstruction coefficient could significantly alter a model’s difficulty score if incorporated, potentially leading to misclassification [34]. Future refinement of the formula should address this issue. Second, our sample size of 19 physicians, while small, is consistent with previous simulation-based studies and provides meaningful preliminary data [35]. Given institutional constraints on participant availability, future multi-center studies could enhance the generalizability of our findings.

Since this is a pilot study, power analysis was not performed, consistent with best practices for feasibility studies [36]. However, based on the observed variability in performance, a future prospective study with an estimated effect size of 0.2952 would require 80 participants across five training levels (16 per group) to achieve adequate statistical power (Supplementary data 1 and 2). Incrementally increasing sample sizes in subsequent studies will allow for more robust validation of our findings.

In summary, this study highlights the importance of simulation-based airway management training and underscores the need for refined manikin designs that better replicate patient anatomy and procedural challenges, which aligns with prior research on airway management training. Patel et al. (2024) demonstrated that independent mastery learning with automated feedback significantly enhances intubation success, reinforcing the importance of structured practice [37]. Yau et al. (2021) highlighted real-time feedback as a key factor in improving proficiency across experience levels [38]. Additionally, Roach et al. (2024) found that different airway simulators improved intubation skills, underscoring the value of simulation-based training [39]. Therefore, our findings provide a foundation for further research into optimizing airway training models to improve intubation success rates and clinical preparedness.

## Conclusions

Our findings confirm that Model C presents the greatest challenge for endotracheal intubation training, as evidenced by its significantly higher difficulty score (4.430), prolonged intubation duration, and increased perceived difficulty. This suggests that Model C may be particularly beneficial for advanced airway management training, equipping trainees with the skills necessary to manage difficult intubations in clinical practice. Additionally, when analyzing physicians across different levels of training in emergency medicine, we found no significant differences in intubation time or success rate, indicating that experience level alone may not be the primary determinant of intubation proficiency. This underscores the importance of structured training protocols and deliberate practice with high-difficulty models, such as Model C, in developing critical airway management skills, rather than relying solely on accumulated years of training.

## Electronic supplementary material

Below is the link to the electronic supplementary material.

Supplementary Material 1

Supplementary Material 2

Supplementary Material 3

Supplementary Material 4

Supplementary Material 5

## Data Availability

Availability of data and materialsThe source code of R can be downloaded from supplementary materials, and the data sets generated during and/or analyzed during this study are available from the corresponding author on reasonable request.

## Abbreviations

OSA: Obstructive Sleep Apnea
BMI: Body Mass Index
IRB: Institutional Review Board
SPSS: Statistical Package for the Social Sciences
CPR: Cardiopulmonary Resuscitation
PGY: Post-Graduate Year
R: Residency (R1, R2, etc., for years of residency)
PoC: Proof of Concept
