# Supplementary figures and images for: Using the predictive model of difficult endotracheal intubation to examine different simulators for airway management training: a pilot cross-sectional observational study
Source: BMC Med Educ. 2025 Jun 6;25:848. doi: 10.1186/s12909-025-07413-2 (PMC12144714; doi:10.1186/s12909-025-07413-2)

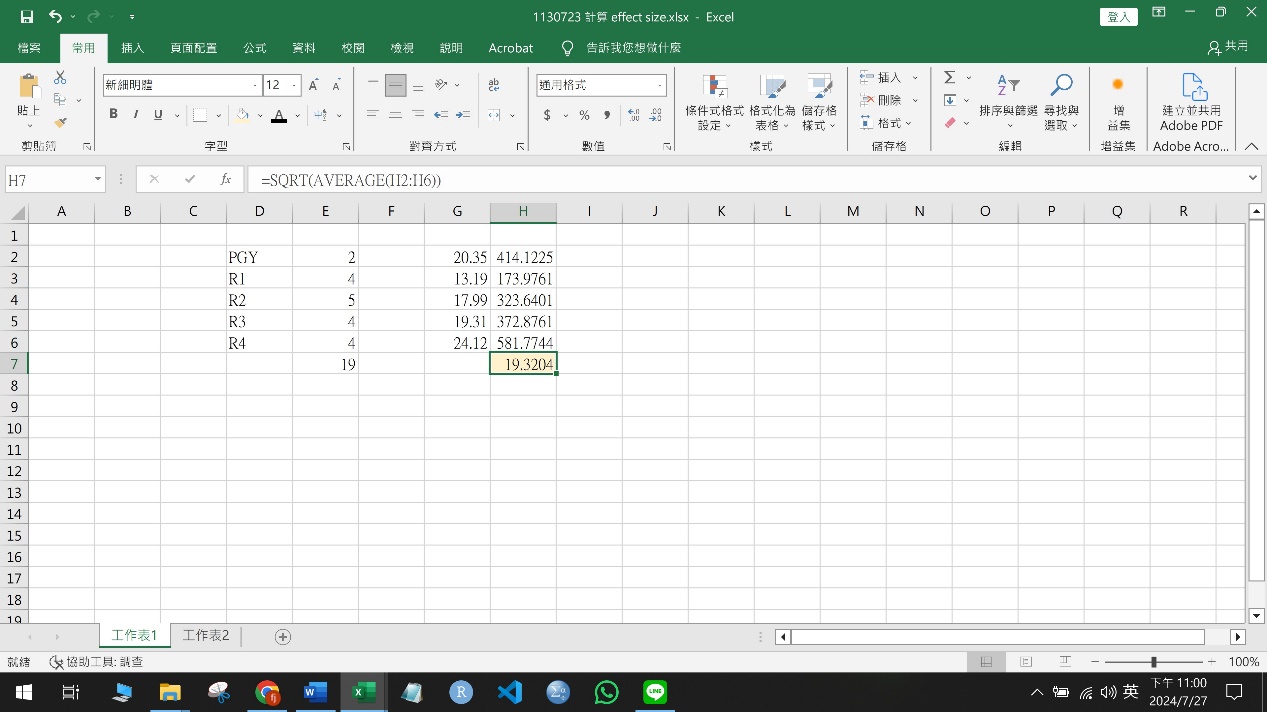

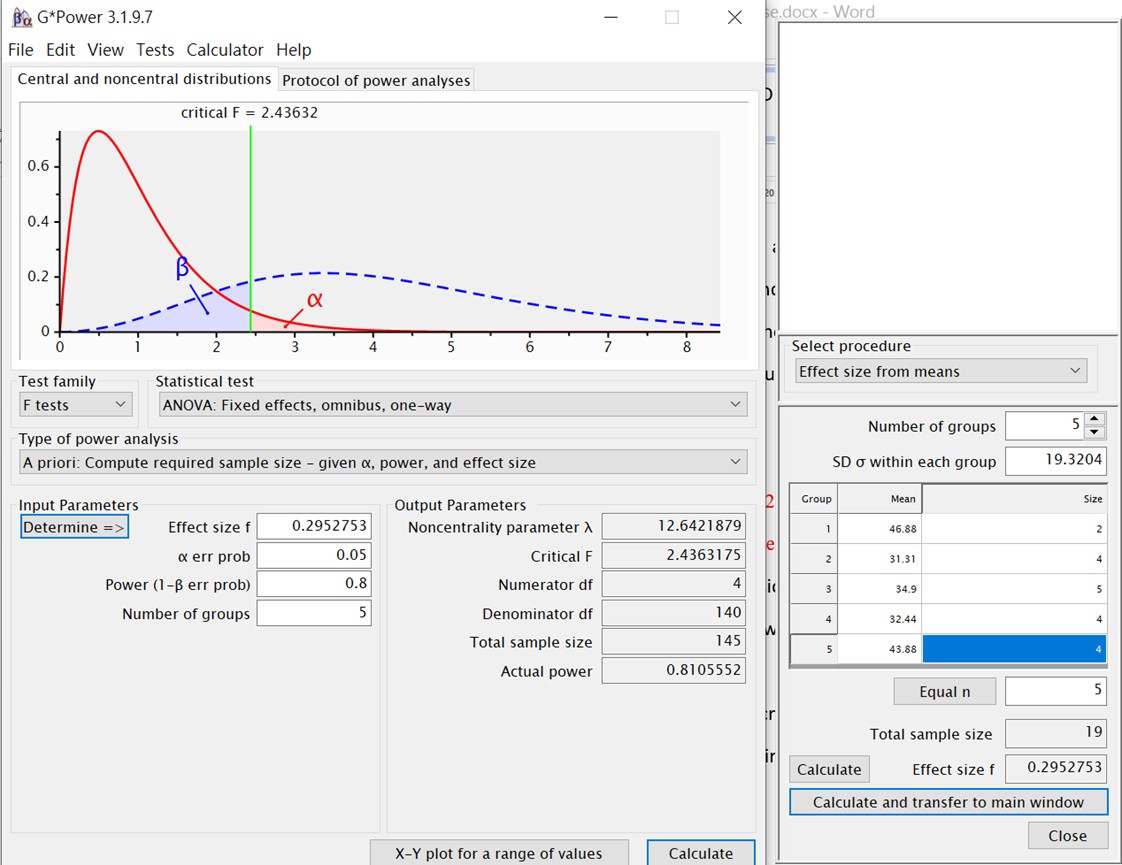

Supplement: Supplementary file 1 — Supplementary Material 1 [file 12909_2025_7413_MOESM1_ESM.docx]
